# Supplementary material for: Single cell transcriptomics identifies a unique adipose lineage cell population that regulates bone marrow environment
Source: eLife. 2020 Apr 14;9:e54695. doi: 10.7554/eLife.54695 (PMC7220380; doi:10.7554/eLife.54695)
Supplement: Supplementary file 1. [file elife-54695-supp1.docx]

| Gene | Forward primer | Reverse primer |
| --- | --- | --- |
| *Cebpa* | 5’-CAAGAACAGCAACGAGTACCG-3’ | 5’-GTCACTGGTCAACTCCAGCAC |
| *Lpl* | 5’-GGGAGTTTGGCTCCAGAGTTT-3’ | 5’-TGTGTCTTCAGGGGTCCTTAG |
| *Adipoq* | 5’-AAAGGAGAGCCTGGAGAA-3’ | 5’-GAATGGGTACATTGGGAACA-3’ |
| *Pparg* | 5’-CCAGCGTGAAGCCAGAGTAG-3’ | 5’-ACCGTGGCTGTGCTCATCCT-3’ |
| *Lepr* | 5’-TGGTCCCAGCAGCTATGGT-3’ | 5’-ACCCAGAGAAGTTAGCACTGT-3’ |
| *Cxcl12* | 5’-CTGTGCCCTTCAGATTGTT-3’ | 5’-AGCTTTCTCCAGGTACTCTT-3’ |
| *Serpina3g* | 5’-CTGTGGTGGAGCTGAAATAC-3’ | 5’-TCAGGGTCTCTGGTTGTAAG-3’ |
| *Agt* | 5’-TCCACTGACCCAGTTCTT-3’ | 5’-AAGTAGGGTGCTGTCTGT-3’ |
| *Gdpd2* | 5’-CAGGAGTGGCATAGTTTACG-3’ | 5’-CAGGCCAACAAGGTGATT-3’ |
| *Il1rn* | 5’-TGCCAAGTCTGGAGATGA-3’ | 5’-CAGAGCGGATGAAGGTAAAG-3’ |
| *Kng1* | 5’-GAGACCTTGGGAGAACAAAG-3’ | 5’-ACACTCCGGAAAGGAGAA-3’ |
| *Kng2* | 5’-GGACTCCTGCTGACTTTAAC-3’ | 5’-GCATCCACAGCCTGAAATA-3’ |
| *Esm1* | 5’-TACAGCGAGGAGGATGATT-3’ | 5’-GGCAATTGCAAGTCTCTTTG-3’ |
| *Tnfsf11* | 5’-CAGCATCGCTCTGTTCCTGTA-3’ | 5’-CTGCGTTTTCATGGAGTCTCA-3’ |
| *Vegfa* | 5’-GCACATAGAGAGAATGAGCTTCC-3’ | 5’-CTCCGCTCTGAACAAGGCT-3’ |
| *Vegfc* | 5’-GAGGTCAAGGCTTTTGAAGGC-3’ | 5’-CTGTCCTGGTATTGAGGGTGG-3’ |
| *Rspo3* | 5’-ATGCACTTGCGACTGATTTCT-3’ | 5’-GCAGCCTTGACTGACATTAGGAT-3’ |
| *Angpt4* | 5’-CAGCCAGCTATGCTACTAGATGG-3’ | 5’-CCTCTGGAGGCTATTGGAGC-3’ |
| *Actb* | 5’-GGCTGTATTCCCCTCCATCG-3’ | 5’-CCAGTTGGTAACAATGCCATGT-3’ |
